# Supplementary material for: Exploring Epitaxial Structures for Electrically Pumped Perovskite Lasers: A Study of CsPb(Br,I)3 Based on the Ab Initio Bethe–Salpeter Equation
Source: Materials (Basel). 2024 Jan 15;17(2):0. doi: 10.3390/ma17020427 (PMC11154405; doi:10.3390/ma17020427)
Supplement: Supplementary file 1 [file materials-17-00427-s001.zip › materials-2707001-supplementary.pdf]

Supplementary Materials

"Exploring epitaxial structures for electrically pumped perovskite lasers: a study of $CsPb(Br,I)_3$ based on the *ab initio* Bethe-Salpeter equation"

Małgorzata Wierzbowska[a,*] and Juan J. Meléndez[c,d]

[a] *Institute of High Pressure Physics, Polish Academy of Sciences*
[c] *Department of Physics, University of Extremadura.*
[d] *Institute for Advanced Scientific Computing of Extremadura (ICCAEX).*
* Corresponding author: wierzbowska@unipress.waw.pl.

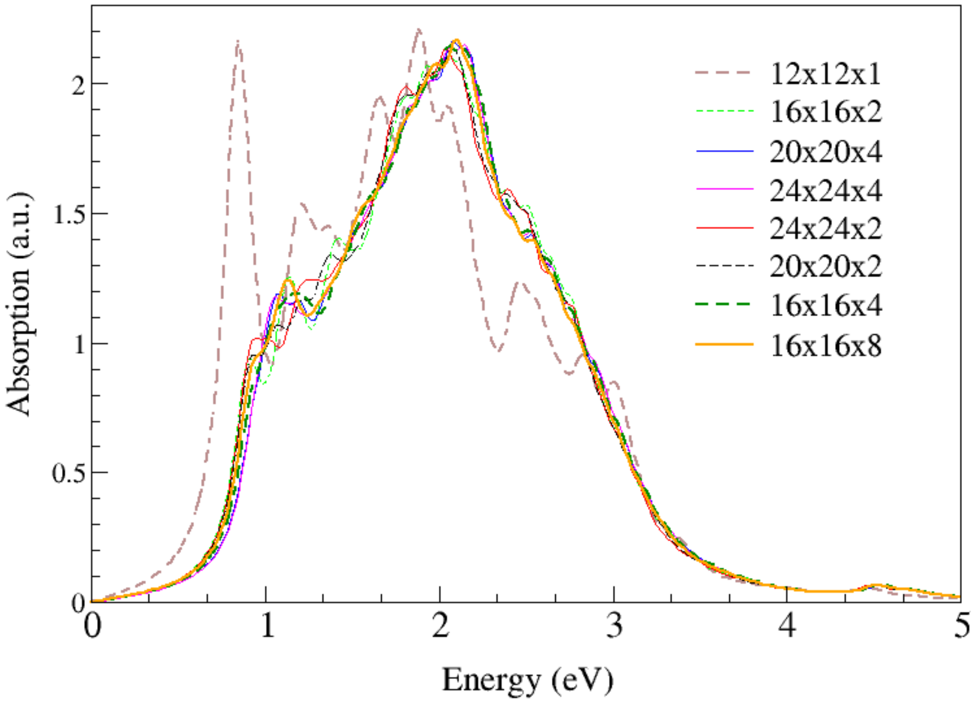

Figure S1: Convergence test for the BSE absorption spectrum for M1 supercell and polarisation [100] performed with respect to the sampling grid in the BZ.

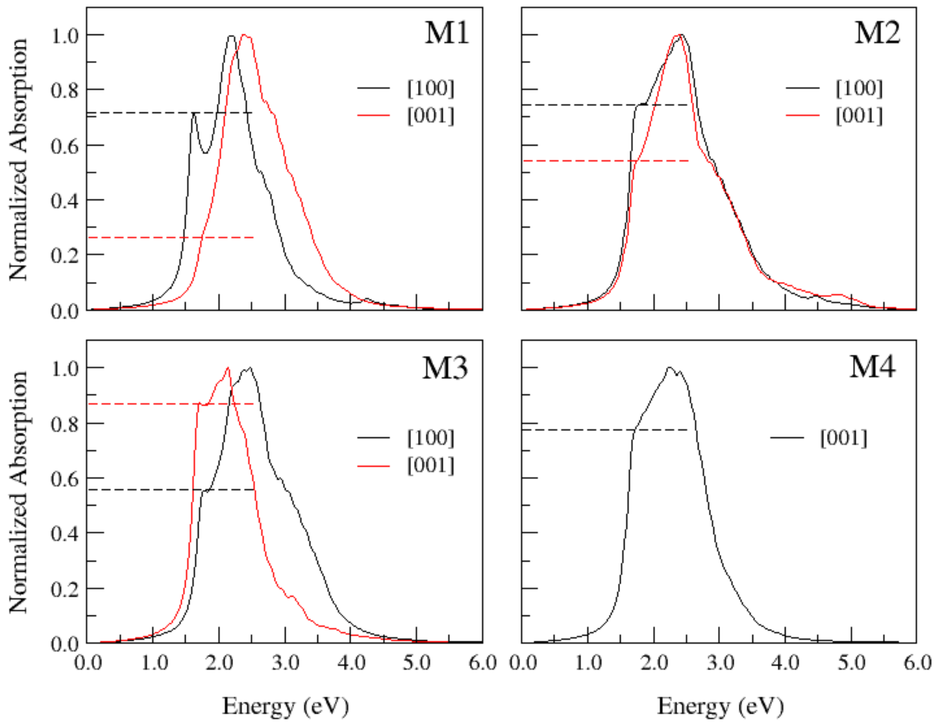

Figure S2: Normalised absorption spectra obtained from the non-relativistic *ab initio* Bethe-Salpeter equation for the supercells considered in this work. In this and the following figure the electric field is chosen either along [100] (in-plane) or along [001] (out-of-plane). The dashed lines indicate the height of the first prominent peaks.

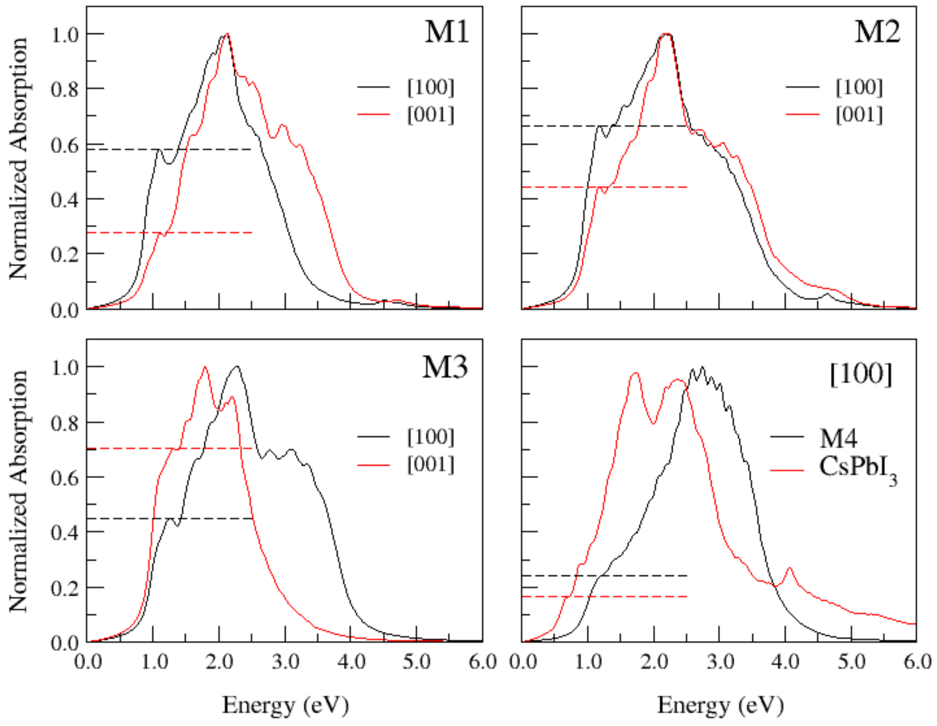

Figure S3: Normalised absorption spectra obtained from the *ab initio* Bethe-Salpeter equation including SOC included for the supercells considered in this work and for pure $CsPbI_3$.

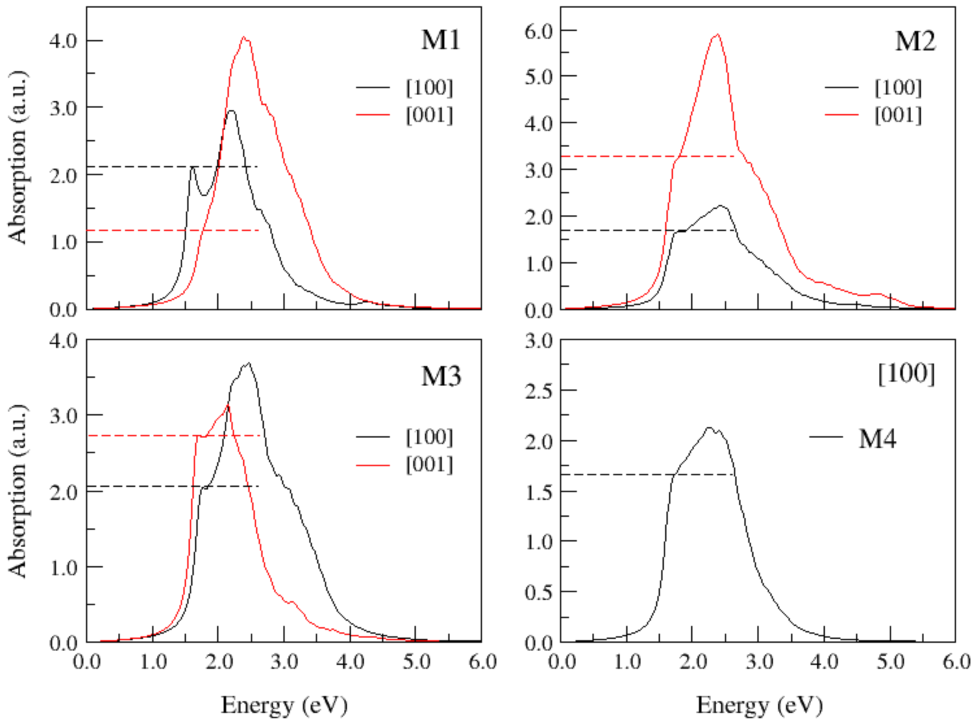

Figure S4: Absorption spectra (not normalised) obtained from the non-relativistic *ab initio* Bethe-Salpeter equation for the supercells considered in this work.
